# Prediction of football injuries using GPS-based data in Iranian professional football players: a machine learning approach

**DOI:** 10.3389/fspor.2025.1425180

**Published:** 2025-01-31

**Authors:** Reza Saberisani, Amir Hossein Barati, Mostafa Zarei, Paulo Santos, Armin Gorouhi, Luca Paolo Ardigò, Hadi Nobari

**Affiliations:** ^1^ Department of Health and Sport Rehabilitation, Faculty of Sport Sciences and Health, Shahid Beheshti University, Tehran, Iran; ^2^ Faculty of Sports, University of Porto, Porto, Portugal; ^3^ Department of Health Sciences, Doctoral Program in Health and Human Motor Skill, University of A Coruña, Coruña, Spain; ^4^ Department of Teacher Education, NLA University College, Oslo, Norway; ^5^ Laboratorio de Fisiología del Esfuerzo (LFE), Department of Health and Human Performance, Faculty of Physical Activity and Sport Science (INEF), Universidad Politécnica de Madrid, Madrid, Spain

**Keywords:** injury prediction, training load, GPS, football, machine learning

## Abstract

**Introduction:**

The study aims to assess and compare the predictive effectiveness of football-related injuries using external load data and a decision tree classification algorithm by unidimensional approach.

**Methods:**

The sample consisted of 25 players from one of the 16 teams participating in the Persian Gulf Pro League during the 2022--2023 season. Player injury data and raw GPS data from all training and competition sessions throughout the football league season were gathered (214 training sessions and 34 competition sessions). The acute-tochronic workload ratio was calculated separately for each variable using a ratio of 1:3 weeks. Finally, the decision tree algorithm with machine learning was utilised to assess the predictive power of injury occurrence based on the acute-to-chronic workload ratio.

**Results:**

The results showed that the variable of the number of decelerations had the highest predictive power compared to other variables [area under the curve (AUC) = 0.91, recall = 87.5%, precision = 58.3%, accuracy = 94.7%].

**Conclusion:**

Although none of the selected external load variables in this study had high predictive power (AUC > 0.95), due to the high predictive power of injury of the number of deceleration variables compared with other variables, the necessity of attention and management of this variable as a risk factor for injury occurrence is essential for preventing future injuries.

## 1 Introduction

Football players face varying weekly demands depending on training intensity, match frequency, and match-related contextual factors such as congested schedules or back-to-back games. These factors significantly influence the accumulation of training load and contribute to injury risk. As noted by recent research (1–3), congested calendars often lead to altered physical demands, higher injury risk, and diminished recovery time for players, which can complicate load management strategies (4). Considering these contextual elements when predicting injuries is essential, as their impact on training load can increase the likelihood of injury occurrence. Our study aims to build upon this knowledge by investigating the acute-to-chronic workload ratio using machine learning, focusing on how these accumulated loads predict injury outcomes. This predictive model could further assist in injury prevention by offering more precise injury risk assessments in various match contexts. Evidence shows that training and competition expose players to injuries, with studies indicating injury rates of 3.7 per 1,000 training hours and 36 per 1,000 competition hours. In other words, each player experiences at least two injuries on average during a season (5). The high incidence of injuries in this popular field emphasises the importance and necessity of managing risk factors.

Today, numerous risk factors for injuries in football have been identified. Generally, injury is a complex multifactorial process categorised into modifiable and non-modifiable risk factors (6). In recent studies, training load has been identified as a modifiable risk factor for injury (6, 7). Some studies have shown that most injuries occur due to high training loads (8, 9). For example, a specific study revealed that lower limb injuries are most strongly associated with total distance covered, high speed, and sprints (10). Therefore, external load, consisting of various variables such as distance covered at different speeds, body load, acceleration, sprint, metabolic power, etc., can indicate training intensity, acting as a monitoring tool to prevent injuries (11–14). GPS-based data has become an integral part of sports technology, enabling the capture of real-time metrics that were previously unattainable (15). while the validity and reliability of these tools for monitoring training loads have been proven (16); Many studies have worked on variables extracted from GPS, but few studies have combined GPS data analysis and machine learning methods. These advancements allow for more precise injury prediction models, offering novel insights into workload-related injury risks.

Many research studies use variables such as the acute-to-chronic workload ratio, accumulated loads, week-to-week load changes, etc., to compare and analyse their data (17–19). It is widely acknowledged that artificial intelligence and machine learning algorithms are precise tools for detection and decision-making in training management and injury risk assessment. However, they have yet to be extensively utilised, and research studies have primarily relied on patterns other than machine learning (20). In recent years, several studies have explored the potential of machine learning or other ways to predict football injuries using a range of datasets, including GPS-based data (8, 9, 14). However, few have applied the prediction of football injuries using GPS data and machine learning algorithms, especially the decision tree classification model in Asia and the Middle East, which allows for more profound pattern recognition in high-dimensional data. Unlike previous researches (9, 14, 21), using the decision tree classification algorithm. Filling a crucial gap in the literature, this study introduces the ability to predict sports injuries in football with two variables: the average total distance covered and the total distance load covered.

Another advantage of using machine learning for modeling is that it does not require predefined relationships between variables, unlike traditional statistical modeling. Predictions can be made without understanding the underlying mechanisms or making prior assumptions about the fundamental relationships between variables or the random process generating the data. Rossi (22) was among the first to use machine learning for injury prediction modeling, while Carey (23) also focused on injury prediction using similar parameters such as Accuracy, Precision, Recall, and AUC. Both studies recommended machine learning-based modeling methods due to their high accuracy and interpretability. Vallance (24) conducted similar research, utilizing various machine learning algorithms for injury prediction modeling, including Random Forest, Support Vector Machines, K-Nearest Neighbors (KNN), Decision Tree classification, and eXtreme Gradient Boosting (XGBoost).

Given these considerations, previous research has mainly focused on examining the relationship between injuries and training loads rather than predicting injuries. Additionally, traditional statistical methods have been primarily used instead of machine learning techniques. It is important to note that machine learning approaches generally provide better predictive accuracy than conventional statistical methods. Despite these limitations, this study aims to determine if sports injuries in football can be forecasted using external load metrics and machine learning models by unidimensional approach. This research aims to assess and compare the predictive effectiveness of football-related injuries using external load data and a decision tree classification algorithm. Our hypothesis suggests that selected external load metrics derived from Global Positioning System (GPS) data in this study, including total distance covered, average distance covered, distances covered at high and moderate speeds, total distance load covered, accelerations, and decelerations, using machine learning classification tree technique based on four performance metrics AUC, Accuracy, Precision, and Recall, can predict sports injuries in professional football layers in Iran with high power (AUC >95%). Here, this hypothesis suggests explicitly that specific external load variables, especially those related to speed reduction, have significant predictive capabilities in detecting injuries.

## 2 Materials and methods

### 2.1 Participants

During the 2022–2023 season of the Persian Gulf Pro League in Iran, players from 5 teams participating in the league, who met criteria including regular use of GPS monitoring at least three sessions per week, entered the study after providing written consent. However, two teams were excluded from the study due to not using the GPS device in 3 training sessions in some weeks, 1 team due to not using a valid injury registration system, and 1 team due to dissatisfaction with continued cooperation. A sample size of *N* = 25 was chosen based on prior studies (14, 25, 26) demonstrating sufficient power analysis. Therefore, this sample size is adequate to minimize the risk of a Type 2 error. Ultimately, they from one team remained, who were prospectively evaluated (age 26.1 ± 1.26 years, mass 77.5 ± 6.7 kg, height 182.1 ± 7.0 cm, and body mass index 23.4 ± 1.4 kg·m^−2^). Participants were selected from all positions except for goalkeepers, and those who had played at least half a season for the team were chosen. In addition to obtaining written consent and necessary permissions from the club, this study adheres to the Helsinki Declaration and has an ethics certificate with ID IR.SSRC.REC.1402.095 provided by the Research Ethics Committee from the Sport Sciences Research Institute of Iran.

### 2.2 External load quantification

To enable geographical (pitch) tracking, all players wore a GPS-equipped vest (10 Hz) 5–10 min before the start of training or matches and immediately removed the vest after the end of the session. Various studies have shown that 10 Hz GPS has high validity and reliability for measuring various training load variables of athletes (27). It is worth mentioning that the GPS receiver used in this study (ST2) belongs to the Smart Tracking Team brand (Biała Podlaska, Poland) and is of the 10 Hz type. It rigorously monitored signal quality, including dilution of precision (DOP) and the number of connected satellites. DOP values consistently stayed below 2.0, showing high positional accuracy. On average, each GPS device connected to 8–12 satellites per session, optimal for reliable outdoor sports data collection (28). Selected variables of external training load extracted from the GPS receiver are defined in Table 1.

**Table 1:** Definition of GPS variables.

Variable	Definition
Total distance covered	Total distance covered above 3 km·h^−1^ in all training or match sessions in a week (km)
Average total distance covered	Average total distance covered in all match or training sessions in a week (km)
Distances covered at high and moderate speeds	Total distance covered at speeds between 19.8 and 30.0 km·h^−1^ in all match or training sessions in a week (m)
Total distance load covered	Total sum of the product of total distance covered and average recorded speed for each training or match session in a week (m^2^·min^−1^)
Accelerations	Total count of all large acceleration increments greater than 4 m·s^−2^ in all training or match sessions in a week (n)
Decelerations	Total count of all small acceleration decrements less than −4 m·s^−2^ in all training or match sessions in a week (n)

### 2.3 Calculation of GPS variables workload

After collecting GPS data for each variable throughout the season, the acute-to-chronic workload ratio is calculated with a 1:3 ratio using the Acute: Chronic Workload Ratio method (9).

Step 1: calculate the acute workload (AW) (9). In this study, we recorded absolute workloads for each week of the 30-week league for each variable to determine the acute workload for each week.

Step 2: calculate the chronic workload (CW) (9). To calculate the chronic workload based on the 1:3 weekly ratio, we computed the average workload for every 3 weeks according to the following formula (9) and considered it a chronic workload.

CW=(AWn−1+AWn−2+AWn−3)×0.333

Step 3: calculate the acute-to-chronic workload ratio (ACWR) (9). To compute this ratio, we divided the acute workload for each week by the average acute workload over the previous three weeks. Therefore, this ratio was obtained for weeks 4–30 based on the following formula:

ACWR=(AWn)/((AWn–1+AWn–2+AWn–3)×0.333)

### 2.4 Injuries data collection

In this study, injury refers to any collision or non-collision-related injuries that prevented a player from participating in at least one training session or match (29). Therefore, all injuries meeting these conditions and recorded by the team physician were included in the study based on the Fuller et al. classification (29). If a player experienced an injury during a training session or match meeting the conditions as mentioned earlier, the corresponding week was considered as the week of injury for the player, even if the player returned to training or match conditions immediately after the injury within the same week.

### 2.5 The decision tree classification model

Our model uses a learned decision tree from other data (which can be part of past data or part of current data as training data) as a decision-making model to infer conclusions about a dependent variable's value (which is represented in the leaves) from observations about an independent variable (which is defined in the branches). This is one of the predictive modelling approaches used in statistics, data mining, and machine learning (20). Decision tree models are called classification trees in which the target variable can take a set of distinct values. In these tree structures, the leaves represent class labels and the branches represent combinations of features that lead to the class labels (20).

In the current study, the standard code available in the SKLearn library was utilised to apply the decision tree algorithm, employing Python 3.11 programming language (30). The predictive variables used are defined in Table 2. Furthermore, the hyperparameters were set to extract the best results from the decision tree model, as shown in Table 3. Finally, a completely random approach was adopted for model training, allocating 80% of the data for model training and the remaining 20% for model testing.

**Table 2:** Variables applied in the decision tree model.

Variable	(Valuation)	(Value explanation)
Player name	[0,24]	To identify the injured player and match the injury with ACWR for the same player, each player was assigned a code.
Injury status (criterion variable)	[0,1]	Absence of injury = 0
		Occurrence of injury = 1
ACWR	[N_n−1_]	Considering the scale of acute to chronic workload ratio and the classes 0 (no injury occurrence) and 1 (injury occurrence), predicting the occurrence or non-occurrence of injury for each week, which is the period between two matches in the league, one of the predictive variables will be the ratio of acute workload to chronic workload, meaning the ratio of the week before the match to 3 weeks prior to it, for each player.
ACWR mean	[N_n−1_]	Considering the scale of acute to chronic workload ratio and the classes 0 (no injury occurrence) and 1 (injury occurrence), predicting the occurrence or non-occurrence of injury for each week, which is the period between two matches in the league, one of the predictive variables will be the mean of the acute to chronic workload ratio, meaning the ratio of the week before the match to 3 weeks prior to it, for all players.

**Table 3:** Hyperparameters tuning.

Model	HayperParameters
Decision tree classifier	1. Impurity measure = gini
	2. Splitter = best
	3. Min samples split = 2
	4. Min samples leaf = 1
	5. Min weight fraction Leaf = 0.0
	6. Random state = 42
	7. Min impurity decrease = 0.0
	8. CCP alpha = 0.0

Our study's scale for the acute to chronic workload ratio, calculation of variables, and injury prediction is weekly (7 days) (9, 19, 23, 31, 32). Therefore, we measure the injury risk for the upcoming week based on the acute to chronic workload ratio variables calculated in the previous week. In this type of scale, it doesn't matter whether the injury occurs at the beginning, middle, or end of the following week. When an injury occurs for a player during a week, the entire week is defined as the injury occurrence week (i.e., class 1) for the algorithm, and the calculation of other variables is not done for that week. For example, when we calculated the acute to chronic workload ratio for each variable with a 7-to-21-day scale, we used a machine learning algorithm to predict the next week's status (injury = class 1, no injury = class 0). The model's results indicate our model's power in predicting the status for the next 7 days, regardless of whether an injury occurs within those 7 days or not.

### 2.6 Model evaluation

The decision tree algorithm was executed separately for each variable to assess the predictive power of sports injury using selected training load variables in this study. Four indicators, namely accuracy, precision, recall, and area under the curve (AUC), were reported for each variable. The performance metrics used to determine and compare the predictive power of the decision tree model for each variable are defined in Table 4 (24).

**Table 4:** Criteria explanation.

Criterion	Calculation method	Explanation	Description
Accuracy	$\frac{Tp\;+\;Tn}{Tp\;+\;Fp\;+\;Tn\;+\;Fn}$	The ratio of the total number of correctly predicted occurrences [1] and non-occurrences [0] of injuries by the model to the total predictions.	How many predictions are correct?
Precision	$\frac{Tp}{Tp\;+\;Fp}$	The ratio of the total number of correctly predicted occurrences [1] of injuries by the model to the total occurrences and non-occurrences of injuries that the model has correctly predicted.	What percentage of the predicted injuries [1] have occurred?
Recall	$\frac{Tp}{Tp\;+\;Fp}$	The ratio of the total number of correctly predicted occurrences [1] of injuries by the model to the total occurrences of injuries that it has predicted correctly or incorrectly.	What percentage of the predicted injuries have been accurately predicted?
AUC	$\mathrm{Rate}\;\frac{Tp}{Fp}$	The ratio of the total number of occurrences [1] of injuries correctly predicted by the model to the occurrences of injuries incorrectly predicted by it.	What is the model's ability and accuracy in detecting and distinguishing between the occurrence [1] and non-occurrence [0] of injuries in percentage?

TP (true positive: refers to the occurrence of injuries that the model has correctly predicted). FP (false positive: refers to the occurrence of injuries that the model has incorrectly predicted). TN (true negative: refers to the absence of injuries that the model has correctly predicted). FN (false negative: refers to the absence of injuries that the model has incorrectly predicted).

The ideal AUC value of 1 for a model indicates the model's highest capability and perfect accuracy in distinguishing between classes (occurrence of injury/no injury in this study). An AUC value of 0.5 suggests randomness in the model, meaning the model's performance does not significantly differ from random guessing. Finally, an AUC value less than 0.5 indicates that the model performs worse than random guessing, typically due to errors in distinguishing between positive (injury occurrence) and negative (no injury occurrence) samples (33).

## 3 Results

After collecting all injury data at the end of the season, the percentage of player injuries based on the type of injury was as follows: 26.7% of injuries were sprains, 13.3% fractures, 13.3% strains, and 13.3% herniated discs. Additionally, 33.3% of injuries were unspecified in terms of type.

**Figure 1:**
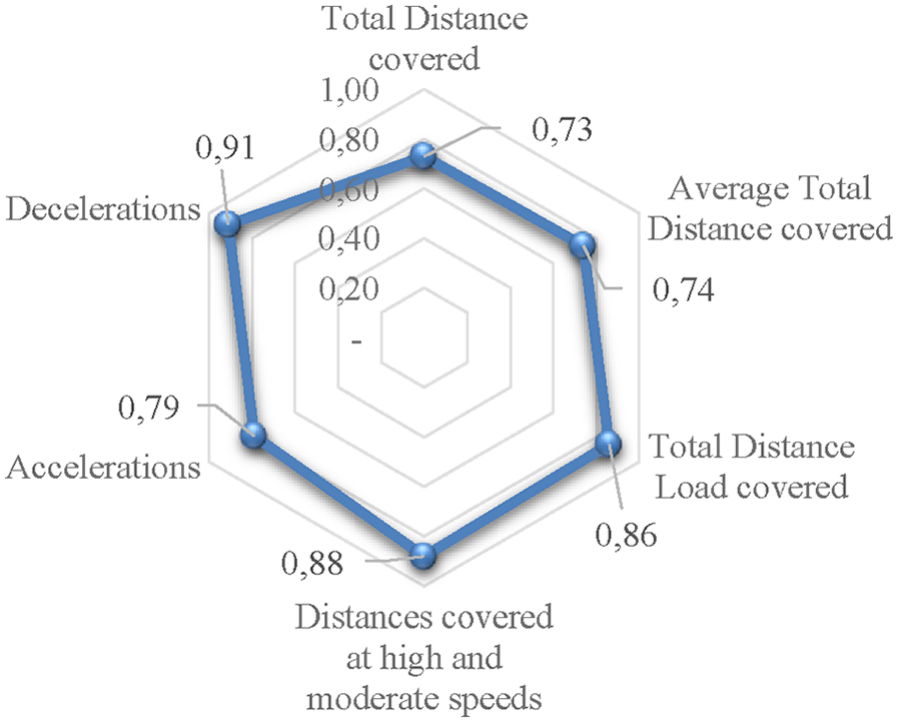
The AUC value of the decision tree model executed on the test data for each variable.

Furthermore, after running the decision tree model seven times for each of the seven external load variables, four selected indices for determining the predictive power of injuries were obtained, with the values of accuracy, precision, recall, and AUC for each of the seven variables presented respectively in Table 5 and Figure 1.

**Table 5:** The accuracy, precision and recall value of the decision tree model executed on the test data for each variable (%).

Variable	Model performance criteria			
	Accuracy (%)	Percision (%)	Recall (%)	Average (%)
Total distance covered	92.9	50	50	64.3
Average total distance covered	93.8	57.1	50	66.97
Distances covered at high and moderate speeds	98.2	100	75	91.07
Total distance load covered	95.6	66.7	75	79.1
Accelerations	95.6	100	58.3	84.63
Decelerations	94.7	58.3	87.5	80.17

## 4 Discussion

This study investigated the predictive power of external load metrics derived from GPS data in Iranian professional football players for the occurrence of sports injuries. The study focused on assessing the feasibility of injury prediction using machine learning techniques, particularly the decision tree classification algorithm. It sought to compare the efficacy of selected external load variables in identifying injury risk. The hypothesis posited that certain external load variables, such as distances covered at high and moderate speeds and the decelerations, would exhibit significant predictive power for sports injuries among football players. The study's main result indicated that, whereas none of the individual variables demonstrated exceptionally high predictive power, distances covered at high and moderate speeds and decelerations emerged as the most influential variables in predicting injury occurrence. These findings contribute to the ongoing discourse on injury prediction in football and offer insights into potential strategies for mitigating injury risk in professional athletes.

Therefore, with a univariate approach, we intend to find suitable and high-powered variables and recommend them for multidimensional approaches. Therefore, in this study, we do not intend to determine values for variables to prevent injury, although past studies have shown that players whose workload ratios fall between 0.8 and 1.0 are exposed to the lowest risk of injury, and if this ratio shifts to either side, the risk of injury increases (31).

Is predicting the occurrence of injury based on training load feasible?

According to our investigations, recent studies in injury prevention are striving to predict sports injuries using various risk factors and machine learning modeling. Different risk factors have been examined so far, including various screening tests (34), data related to three-dimensional motion analysis (35), injury history and muscle strength, and demographic information (36), psychological and neuromuscular risk factors (37, 38), and of course, external load data based on GPS and internal training load (22–24, 39); Regardless of whether each of these studies aimed to predict specific sports injuries (e.g., ankle, hamstring, knee, lower limb, or overall musculoskeletal injuries), all utilized machine learning modeling. However, a noteworthy point is that none of these studies have been able to design a model with high predictive power for injury (AUC >.95). The main limitations of the aforementioned studies have been sample size, limited data, and high risk of bias. Some studies have even suggested that injury prediction may not be feasible (23, 40). However, by focusing on studies that attempt to predict injuries through training load data and rolling averages method, they have only increased prediction accuracy to some extent by adjusting the time intervals of acute and chronic loads and modifying the acute-to-chronic workload ratios. For example, in a study by Carey et al., it was shown that acute-to-chronic workload ratios of 3 or 6 days to 21 or 28-day chronic loads are the best predictors of injuries occurring 2–5 days in the future compared to other ratios (31). In other studies, efforts have been made to increase prediction accuracy by combining internal and external training load variables and using multidimensional approaches and machine learning techniques. However, even studies using multidimensional approaches have not yet found a high level of predictive power for their models (23); Therefore, relying on current scientific knowledge, it cannot be definitively stated that sports injuries are highly predictable through machine learning prediction models.

How can we increase the predictive power of injury prediction models?

Overall, methods for enhancing the predictive power of models can be broadly categorized into two groups: those related to statistical analysis of data and those about variable selection. In the first approach, researchers typically analyze their dataset with multiple machine learning models or traditional statistical models (23, 24, 34, 35) or multiple datasets, which are tuned based on various time intervals (19, 31, 32, 41, 42) or classification of specific injuries, such as hamstring strains (36, 37). They evaluate and analyze them with an evaluation model to find the best and most accurate models in injury prediction, aiming to achieve the highest predictive power. In the second approach, researchers endeavor to achieve the highest predictive power by selecting the most sensitive risk factors for injury, such as variables related to physical readiness (43), results of screening tests (34, 37, 38), three-dimensional motion analysis data (35), neuromuscular measurements (36), and internal and external training loads (22–24, 39, 44), and applying them in their multidimensional models. In this regard, studies have identified numerous and diverse variables as indicators of internal and external training loads, making it impossible to include all of them in a predictive model. Therefore, current studies should move towards selecting the most sensitive variables to injury so that a model with the highest accuracy can be designed. In the path of the second approach to enhancing the power of injury prediction models, due to the recent inclination towards machine learning algorithms and the use of multidimensional models, we decided to select six commonly used external load variables, as discussed previously, with a ratio of 7–21 days, aiming to identify the strongest and most sensitive predictors of injury using a unidimensional approach, as a step towards increasing the predictive power of future studies.

There are various methods and statistical indicators available to determine the predictive power. However, one of the most used indicators that determines the predictive power of predictive models in machine learning algorithms is the AUC metric. Although there may be challenges in determining the predictive power due to limitations such as insufficient data, it is recommended to consider metrics such as accuracy, precision, F1 score (viz., the harmonic mean of the precision and recall values), and especially recall. However, various studies have widely used and accepted the AUC metric, particularly in assessing decision trees’ predictive power and discrimination. In our study, based on the AUC metric, the variable “deceleration” exhibited the highest predictive power.

Nevertheless, considering all four metrics (accuracy, precision, recall, and AUC), the variable “Distances covered at high and moderate speeds” demonstrated the highest prediction accuracy compared with other variables. However, due to our study's limited recorded data on distances covered at high speeds, we combined distances covered at high and moderate speeds under one variable. However, it should be noted that in a study where statistical methods other than machine learning were employed, it was also revealed that the variable “distances covered at moderate speeds” had the highest accuracy in distinguishing between low-risk and high-risk players (relative risk = 2.3–2.6) (31). Nevertheless, another study that aimed at predicting injuries using a multidimensional approach and machine learning and utilised variables such as distances covered at moderate (18–24 km·h^−1^) and high speeds (24 km·h^−1^ and above) separately in the prediction model, it demonstrated limited capability and weak predictive power for the model (AUC <0.65) (23). However, as mentioned earlier, one potential explanation for this discrepancy could be the influence of other variables present in the model, which includes total distance covered, player load, and perceived exertion rank in each session. It was found in the study by Mohr et al. that the rating of perceived exertion (RPE) variable in each session had weak predictive ability for injury (45). Our study also showed that the total distance covered variable had lower predictive power than other variables (AUC = 0.73). Although an AUC value of 0.73 might be considered relatively high compared with other studies, this is likely justified by the limited injury data available. When injury data is scarce, the prediction of injury occurrence may be exaggerated or underestimated. Thus, this predictive capability should be compared with other variables under similar conditions.

Based on our information, limited studies like ours use machine learning algorithms and single-dimensional models. One study that had the most resemblance to our study was by Pilka et al. (46). In this study, the acute-to-chronic workload ratio for training and competition data was calculated using a 7–28-day ratio. Additionally, the study utilized a machine learning algorithm called XGBoost. One notable difference between this study and others is the multidimensional approach to injury prediction. However, in this study, the importance percentage of each variable was also reported separately. The selected variables of decelerations, accelerations, total distance covered, distance covered at high speed, consistent with the variables of our study, and sprint variables, total player load, and field time were new variables compared to our study used in this study. The results of the multidimensional XGBoost model in this study, which had the highest accuracy among the three models in this study, were as follows: Accuracy = 90.0%, Precision = 92.0%, Recall = 97.6%, and F1-Score = 94.7%. According to one of our evaluation methods, namely the interaction of 3 parameters Accuracy, Precision, and Recall, the predictive power of this model was equal to 93.2%, which was considered a high predictive power compared to similar studies. One of the reasons for the high predictive power of this model was the use of deceleration and distance covered at high-speed variables, our study also showed that these variables have high predictive power in a unidimensional approach and their use in multidimensional models will increase their predictive power. Another reason was predicting non-contact injuries, unlike our study, which predicted both contact and non-contact injuries.

In another study conducted by Guitart et al. (47), the aim was to estimate the injury rate based on external training load variables, including total distance covered, distance covered at high speed (>21 km·h^−1^), distance covered with high metabolic load, total time spent in training and competition and player load. The results showed that the highest injury rate was recorded on the competition day and the third day before the competition. It was also evident that the highest training load variables were recorded on these two days. Although the acute-to-chronic workload ratio was not calculated in this study, the results showed that the increase in injury rates was consistent with the increase in absolute values of GPS variables. Although this study, like ours, did not separately calculate the number of accelerations and decelerations, based on the formula for calculating the distance covered with high metabolic load, this variable, derived from the distance covered at a constant speed of 5.5 m·s^−1^ or accelerations and decelerations, indicates that this variable indirectly considers both acceleration and deceleration simultaneously and is their resultant. In this regard, our study also showed that, apart from player load and total time spent in training and competition, which were not measured, the remaining variables also had at least moderate predictive ability for injuries. However, the separate examination of accelerations and decelerations showed that decelerations had a higher predictive ability for injuries than accelerations.

However, the study by Bacon et al. (48) showed different results than ours. Although this study used statistical methods other than machine learning, the aim was to investigate the predictive ability of two variables: total distance covered and distance covered at high speed in predicting overuse injuries. The results of this research indicated that the total distance parameter had a significantly higher predictive ability (F_1,39_ = 6.482, *p* = 0.015) compared with the distance covered at high-speed parameter (F_1,39_ = 1.003, *p* = 0.323) and it could effectively impact the occurrence of football players’ overuse injuries. The comparison of these two variables in our study showed contradictory results, indicating that the variable of distance covered at moderate and high speeds (accuracy = 98.2%, precision = 100%, recall = 75%, AUC = 0.88) had significantly higher predictive ability compared with the total distance variable (accuracy = 92.9%, precision = 50%, recall = 50%, AUC = 0.73) and influenced most of the players’ sports injuries. In presenting these contradictory results, it should be noted that in our study, the integration of collision and non-collision injuries into the outcome variable and the integration of variables of distance covered at high speed and distance covered at moderate speed in one predictive variable did not seem to affect the presentation of results. Additionally, our investigations showed that Iranian Premier League players’ distance covered at high speed is lower than that of players in reputable European leagues. This factor could also be another influential factor in comparing the results.

With these interpretations, the evidence suggests that distances covered at high and moderate speeds and decelerations are appropriate variables with high predictive power and will increase the AUC of multi-dimensional predictive models. However, in contrast, the total distance covered and its average may be inappropriate variables and potentially lead to a decrease in the model's predictive power.

### 4.1 Limitations

Some of the most significant limitations of the current study include a small sample size and a short follow-up period (one season). Other limitations worth noting include the failure to investigate other GPS variables such as total training and competition time, player load, metabolic load, and distances covered as external load variables, and RPE as an internal load. Furthermore, the scarcity of injury data can be highlighted as a primary limitation of the current study. Additionally, the rarity of recorded injuries led to the inability to accurately classify and utilize contact and non-random injuries as a single injury. Distinguishing between these two types of injuries and classifying recorded injuries based on player stoppage location and duration may improve model performance. Another limitation of this study was that we did not have access to detailed injury data, which made us not have access to clinical records; This limitation limits us to provide accurate diagnoses, types and severity levels of injuries. Besides the limitations above, the present study was conducted on a team from the Iranian Premier League using the Smart Tracking Team brand GPS model (Biała Podlaska, Poland), and only one machine learning model was used in this study. These factors should be considered when generalizing the results to other contexts.

### 4.2 Practical implications

This study serves as a guideline for future similar studies, especially those aiming to predict sports-related injuries in football using multidimensional approaches. Therefore, in the first step, we recommend utilizing variables such as decelerations and distances covered at high and moderate speeds in their multidimensional models due to their high predictive power. Incorporating these variables into their models is likely to enhance their predictive power, given their relatively high predictive power. In the second step, this study has practical applications for injury risk management during intensive weeks of professional football. Therefore, it is recommended that coaches and team analysts regularly monitor the ratio of acute, chronic, and acute-to-chronic workload imposed on athletes, especially regarding variables such as decelerations and distances covered at high and moderate speeds. This monitoring could increase the time between acute injuries and reduce the overall number of acute injuries among players throughout the season. While this study provides valuable insights, limitations include the sample being restricted to one league. Future research should explore cross-league datasets to assess the model's applicability across different playing conditions. Additionally, psychological and environmental factors could enhance the model's predictive power.

## 5 Conclusion

The findings of this study indicate that none of the individual variables possess high predictive power on their own. However, while the variable of deceleration exhibited the highest predictive capability based on one of the key performance indicators of the model (AUC), the distances covered at medium and high speeds demonstrated the most excellent overall predictive power when considering all four performance indicators collectively. This suggests that a multifaceted approach, integrating various performance metrics, is essential for enhancing injury prediction accuracy in football players.

Therefore, based on the results, while recommending the incorporation of these two variables in future studies to enhance the predictive power of multidimensional injury prediction models, it is suggested that coaches primarily focus on the variable of distances covered at high and moderate speeds and secondarily on the variable of decelerations, as the strongest injury predictors. They should pay special attention to these variables throughout the season to prevent sudden increases in the acute-to-chronic workload ratio and prevent sports injuries.

## Data Availability

The raw data supporting the conclusions of this article will be made available by the authors, without undue reservation.
